# Effects of Propofol General Anesthesia on Olfactory Relearning

**DOI:** 10.1038/srep33538

**Published:** 2016-09-15

**Authors:** Li-Jie Jia, Pei Tang, Nicole R. Brandon, Yan Luo, Buwei Yu, Yan Xu

**Affiliations:** 1 Department of Anesthesiology, University of Pittsburgh School of Medicine, Pittsburgh, PA 15213, USA; 2 Department of Anesthesiology, Ruijin Hospital, Shanghai Jiao Tong University School of Medicine, Shanghai, 200025, China; 3 Department of Pharmacology and Chemical Biology, University of Pittsburgh School of Medicine, Pittsburgh, PA 15213, USA; 4 Department of Computational and Systems Biology, University of Pittsburgh School of Medicine, Pittsburgh, PA 15213, USA; 5 Department of Structural Biology, University of Pittsburgh School of Medicine, Pittsburgh, PA 15213, USA.

## Abstract

How general anesthesia interferes with sensory processing to cause amnesia remains unclear. Here, we show that activation of a learning-associated immediate early gene in rat olfactory cortices is uninterrupted by propofol, an intravenous general anesthetic with putative actions on the inhibitory GABA_A_ receptors. Once learned under anesthesia, a novel odor can no longer re-activate the same high-level transcription programming during subsequent conscious relearning. Behavioral tests indicate that the animals’ ability to consciously relearn a pure odorant, first experienced under general anesthesia, is indeed compromised. In contrast, when a mixture of two novel odorants is first experienced under anesthesia and then relearned consciously in pairs with one of the components, the animals show a deficit in relearning only the component but not the mixture. Our results reveal a previously unknown mechanism of unconscious memory due to irreplaceable neuronal commitment under general anesthesia and support the notion that general anesthesia acts at stages beyond cellular coding to disrupt sensory integration for higher-order association.

We showed recently that deeply anesthetized rats could still respond to novel odors by altering the immediate early gene activation in the anterior piriform cortex and orbital frontal cortex, the two higher-order olfactory processing centers, but could not behaviorally remember the experience^1^. This intriguing phenomenon suggests that general anesthesia does not completely block sensory processing, but nevertheless disrupts the integrity of the associated memory, preventing it from being adequately retrieved. In bees and flies, it has been demonstrated that when an odor-corresponding region is occupied or committed by being associated with a positive stimulus, the same region cannot be released for the same odor to act as a negative stimulus^2^,^3^. If a similar mechanism operates in mammalian brains, one would expect that an ensemble of neurons already committed to code a given odorant under general anesthesia cannot be readily modified to re-code the same odorant consciously. Consequently, the ability to relearn the same odorant that has been first experienced under general anesthesia will possibly be impaired.

To test this hypothesis, we created an olfactory learning-relearning paradigm (Fig. 1), in which rats were subjected to an initial exposure to a novel odorant either awake or under a surgical plane of general anesthesia (learning) and then re-exposed to the same odorant without anesthesia 24 h later (relearning). Separate animals in parallel experiments were evaluated histologically for the activation of neuronal PAS domain protein 4 (Npas4) and behaviorally for odor memories using the forced-choice recognition test^1^,^4^. Npas4 is a recently discovered sensory-activity-dependent transcription factor known to initiate experience-dependent learning and memory through the regulation of inhibitory synapses on excitatory neurons^5^, and the forced-choice recognition test measures animals’ familiarity–a component of recognition memory^6^,^7^–with the experimental odorants. In this study, we used isoamyl acetate and decanal as the pure odor stimulants in a binary (novel versus familiar) design. Previous studies with multiple odorants showed that rats had an equal and favorable preference for isoamyl acetate and decanal in behavioral testing^1^. We found that olfactory activities continued to alter memory-related transcriptional programming under general anesthesia, leading to neuronal commitment to specific odorants. This commitment is not modifiable later during conscious relearning. Such an “unconscious cellular memory”, while irretrievable, was nevertheless manifested as impairment to relearning the anesthesia-paired odorant, suggesting that general anesthesia may potentially affect the formation of future memories. Moreover, we also found that the association of two novel odorants, but not the individual components, could escape such relearning deficits, indicating that integration and consolidation of sensory information did not occur under general anesthesia and remained accessible for subsequent conscious modulation.

## Results

### Propofol does not block transcriptional programming for experience-dependent learning and memory

We first compared the olfactory cortex responses to novel odor stimulations under fully awake and fully anesthetized conditions by quantifying the Npas4^+^ neurons in the anterior piriform cortex, a high cortical region to which olfactory sensory inputs converge. Npas4^+^ immunoreactivity in Layer II of the anterior piriform cortex was quantified by calculating the percentage of Npas4^+^ pixels in total pixels in the region of interest using segmentation analysis of the dot plots from the Npas4-DAPI doubly stained micrographs. Rats were randomized into five different groups: (1) control; (2) learning a novel odor while awake; (3) learning a novel odor while fully anesthetized; (4) learning a novel odor while awake and relearning the same odor while awake 24 h later; and (5) learning a novel odor fully anesthetized and relearning the same odor while awake 24 h later. For the anesthetized groups, a surgical plane of general anesthesia was achieved and maintained with propofol, a clinical intravenous general anesthetic with a primary action on the inhibitory GABA_A_ receptors. Rats in the awake groups were handled in the same way as the propofol-anesthetized animals but received an intraperitoneal injection of normal saline. Compared to the control animals, which were handled similarly but did not receive novel odor stimulation, animals exposed to a novel odorant elicited significantly higher levels of Npas4 expression 1 h after the odor exposure (Fig. 2A,B). Table 1 summarizes the medians and the 95% confidence intervals (CI) of the Npas4^+^ immunoreactivities that are two standard deviations (2σ) above the mean background Npas4^+^ fluorescence intensity. After the immunoreactivity data were ranked for normality and equality of variance, one-way analysis of variance (ANOVA) with *post hoc* pairwise comparisons showed that the experience-dependent activation of Npas4 was not significantly different between the awake and propofol-anesthetized groups (Fig. 2C, mean rank difference = 1.64, p = 0.508, 95% CI [−4.62, 7.28]), suggesting that the novel odor exposure triggered a similar level of Ca^2+^-influx-mediated modification of the experience-dependent transcriptional programming^8^,^9^,^10^, even when the animals were fully anesthetized. Thus, in excellent agreement with our previous results obtained under ketamine/xylazine general anesthesia^1^, cellular “learning” of a novel odorant continues to occur under propofol general anesthesia, resulting in learning-associated immediate early gene activation in the higher olfactory cortex.

### Cellular coding of an odorant under propofol anesthesia prevents subsequent conscious re-coding for the same odorant

The physical “imprint” of odor exposure, manifested as the Npas4 activation in the anterior piriform cortex, also affects the subsequent experience-dependent transcription programming. For the relearning groups, the same odorant, given one day later to the same animals while awake, triggered a significantly lower level of Npas4 activation (see Table 1 and Fig. 2B,C), indicating that the same odorant was no longer regarded as novel in the sensory process, even when the previous exposure was given under deep anesthesia. The mean rank differences in Npas4^+^ immunoreactivity are (Mean [95% CI]): 16.4 [11.1, 22.0], 17.3 [11.5, 23.7], 18.0 [13.4, 22.3], and 18.9 [13.7, 24.5] between Groups 2 and 4, Groups 2 and 5, Groups 3 and 4, and Groups 3 and 5, respectively (all p values <0.001). The decreased overall Npas4 activation during the second exposure (Fig. 2C) also suggested that the same set of neurons, instead of a completely different set, was involved in re-coding the same specific odorant during conscious relearning.

### Propofol blocks both cognitive memory and future relearning

We next examined the behavioral consequences related to the experience-dependent Npas4 expression. As shown above, the first exposure to a novel odorant triggered a high-level Npas4 activation and the second exposure to the same odorant failed to re-activate the same level of Npas4 expression, irrespective of whether the first exposure was under an awake or fully anesthetized condition. Although both results indicate possible transcriptional programming for odor-associated learning and memory during the first exposure, the animals in the anesthetized group were nevertheless unable to behaviorally recognize the odorant experienced under anesthesia as familiar. Specifically, with forced-choice recognition tests, we contrasted two different novel odorants, isoamyl acetate and decanal, as Odor A and Odor B in a counter-balanced, randomly interchanged fashion for different animals. Although these two experimental odorants elicit the same level of curiosity among rats in smelling behavior^1^, the counter-balanced design cancels any residual preference bias so that differences in smelling time will provide a sensitive measure of cognitive memories about the experimental odors. Figure 3A summarizes the results from initial learning. The control animals showed equally strong interest towards both Odor A and Odor B (median difference, MD = 0.009, p = 0.834, 95% CI [−0.024, 0.047]) because this group had never been exposed to either odorant before. If the initial learning was under the awake condition with Odor A and behavioral testing was done 24 h later alongside a different novel odorant (Odor B), the animals showed a preference for Odor B (MD = 0.363, p = 0.047, 95% CI [0.042, 0.497]) because the animals regarded Odor A as familiar. If Odor A was “learned” under propofol anesthesia, the animals showed equal preference for Odor A and Odor B (MD = 0.088, p = 0.873, 95% CI [−0.014, 0.164]), as if the anesthesia-paired odorant (Odor A) were experienced for the first time. Clearly, propofol altered the sensory processes to such an extent that the animals in the propofol group were unable to later remember having experienced the odorant under anesthesia.

**Figure 1:**
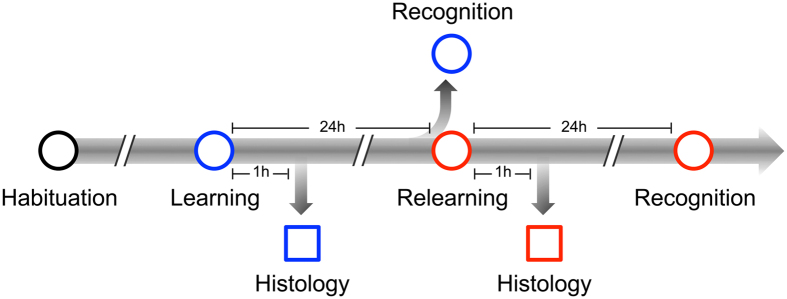
Schematic of the learning and relearning protocol. After habituation of 20 min per day for 3 days, animals in different groups were subjected to odor learning (blue) and relearning (red) with and without anesthesia. Circles indicate placement of animals in the behavioral testing chamber for 20 min, the first 5 min of which were recorded and quantified for odor-smelling behavior. Squares indicate termination of subsets of animals for histology measurements. Learning involves exposure to either a pure odorant (Odor A) or a mixture of two pure odorants (Odor AB). Relearning involves exposure to the same odorant(s) alongside either a different novel odorant (Odor B) or one of the components in the mixture. Behavioral tests involve forced-choice recognition among four beads, two of which are scented with experimental odorants (Odor A, Odor B, or Odor AB) and the remaining two are unscented background beads from the animals’ home cage.

**Figure 2:**
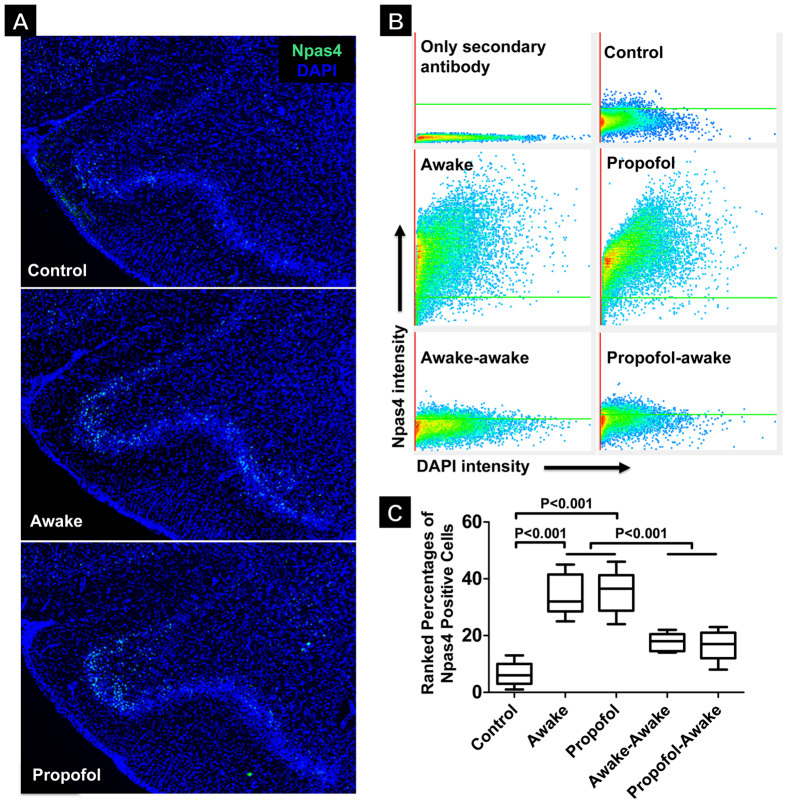
Learning-associated transcriptional programming as revealed by Npas4 activation. (**A**) Representative transverse sections of Npas4 staining (green) in the anterior piriform cortex (APC). (**B**) Scatter plots of fluorescence intensities for Npas4 and DAPI immunostaining in Layer II of APC under various conditions. The green lines mark two standard deviations (upper ~95% confidence interval) above the mean Npas4 background fluorescence in the control group. Cells with fluorescence intensity above this threshold are considered Npas4 positive. (**C**) Quantification by percentage of Npas4^+^ cells in Layer II of APC (Box and whisker show the median, 25^th^ and 75^th^ percentiles, and the full data range; n = 5–14 sections from three rats in each group). The *P* values were derived from analysis of variance on the ranked data with *post hoc* pairwise comparisons. Control, animals that were handled identically to the experimental groups but received no odor stimulations; Awake, animals that learned a novel odorant for 20 min under a fully awake condition; Propofol, animals that were exposed to a novel odorant for 20 min after fully anesthetized by propofol; Awake-awake, animals that learned a novel odorant while awake and then relearned the same odorant while awake 24 h later; and Propofol-awake, animals that were exposed to a novel odorant under propofol anesthesia and then relearned the same odorant while awake 24 h later.

**Figure 3:**
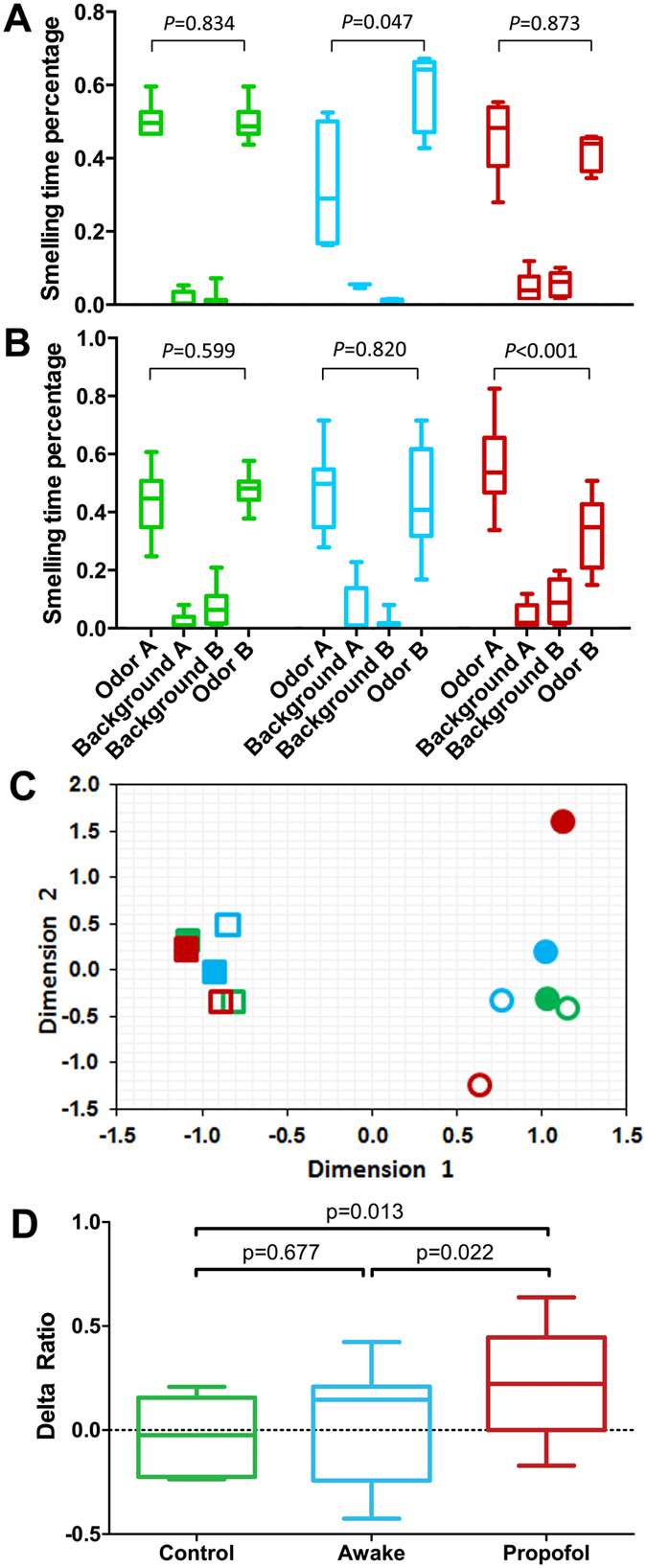
Forced-choice memory tests after learning and relearning a pure odorant. (**A**) Percentage of time spent smelling each of four beads is normalized against the total smelling time during the first recognition test depicted in Fig. 1 (*i.e.*, after initial learning). For the Control (green), Awake (cyan), or Propofol (red) group, Odor A was novel, previously learned under the awake condition, or previously presented under propofol anesthesia, respectively. Odor B was novel to all groups during this phase of behavioral testing. Background A and Background B were two beads from the animals’ home cage as a familiarity reference. (**B**) Percentage of time spent smelling each of the four beads during the second recognition test in Fig. 1 (after relearning). Group colors are the same as in (**A**). Odor A was used in the initial learning for the awake and propofol groups, and both Odor A and Odor B were used in the conscious relearning for all groups. (**C**) Correspondence analysis of the discretized data from (**B**). Color scheme is the same as in (**A**,**B**). Solid and open circles are Odor A and Odor B, respectively, and solid and open squares are Background A and B, respectively. (**D**) The delta ratios were calculated from (**B**) as (Odor A − Odor B)/(Total Smelling Time). Propofol-induced relearning deficit is clearly indicated as a significantly higher percentage of time spent on the odorant initially learned under anesthesia. Box and whisker plots display the median, 25^th^ and 75^th^ percentile, and the full range from n = 6–12 rats per group. The *p* values were obtained from pairwise Kruskal-Wallis non-parametric test for (**A**) and (**B**), and ANOVA with least significant difference post hoc test for (**D**).

The relearning procedure in this study involved letting the animals experience a novel odorant (Odor A) either awake or under propofol anesthesia (initial learning) and then relearn the same odorant 24 h later along with a competing novel odorant (Odor B) in a fully conscious state (see Fig. 1 and the legend). Thus, the animals were exposed to Odor A twice but Odor B only once. The second forced-choice recognition tests (Fig. 3B) showed that the control and awake groups again displayed equal preference for the two odorants (MD = 0.044, p = 0.599, 95% CI [−0.032, 0.225]; and MD = 0.146, p = 0.820, 95% CI [−0.146, 0.182], respectively). Irrespective of the odorant identity in the counter-balanced design, animals in the propofol group always spent more time with Odor A (MD = 0.223, p < 0.001, 95% CI [0.010, 0.340], Fig. 3B) as if it were less familiar or more interesting. After the percentage of smelling time is discretized by binning, the correspondence analysis shows clear clustering of the times spent on background odors, which are separated from the times spent on Odor A and Odor B (Fig. 3C). Most importantly, for the propofol group, the data for Odor A and Odor B are completely separated into two distinct clusters. The delta ratios (Fig. 3D), which quantify the animals’ cognitive memory towards the two experimental odorants, showed no significant difference from zero for the Control group (median = −0.024, 95% CI [−0.146, 0.100]) and the Awake group (median = 0.146, 95% CI [−0.195, 0.197]), but are significantly different from zero for the Propofol group (median = 0.223, 95% CI [0.010, 0.340]). Moreover, pairwise comparison showed that the Propofol group is significantly different from the Control (Mean Difference = 0.250, p = 0.013, 95% CI [0.093, 0.415]) and Awake group (Mean Difference = 0.210, p = 0.022, 95% CI [0.025, 0.399]). Clearly, the animals in the Propofol group showed a deficit in their ability to remember previous experiences with Odor A, despite the fact that Odor A was presented twice. This result is consistent with the notion that olfactory regions associated with coding Odor A under general anesthesia cannot be released to reactivate learning-associated Npas4 by the same odorant during the second conscious exposure (Fig. 2), thereby impeding the animals’ ability to relearn and memorize Odor A.

### Learning an odor mixture is more than just coding its components

It is worth noting that the impaired ability of the anesthetized animals to later reinforce an unconsciously acquired “memory” – if it is indeed acquired – is a previously unknown phenomenon that is unrelated to memory repression or suppression^11^. The results illustrate a type of drug-induced future memory blockade that is not only specific, but also selective. In order to further analyze this phenomenon, we investigated whether the integrated experience of smelling a mixture of two odorants can be distinguished from the experience of smelling one of the components in the mixture. It has been well established that different pure odorants are coded by different combinatory sets of olfactory receptors, from which the sensory inputs unique to individual odorants are mapped separately onto spatially segregated areas in the olfactory bulb^12^,^13^ and then projected to the anterior piriform cortex. Using two structurally distinct odorants in an odor mixture, we expect that the odor coding for the two components should reach the olfactory cortex through different projection pathways. Given that propofol anesthesia impairs relearning of a pure odorant, as shown in Fig. 3B,D, we ask (1) whether relearning of individual components in an odor mixture is also impaired, assuming that the physical coding of the two components in the mixture occurs independently of each other under anesthesia; and (2) whether the experience of smelling the mixture evokes more than just the coding of individual components. The rationale is that if the experience of smelling the mixture can be fully captured by coding individual components, then relearning the mixture should be as impaired as for its components. Otherwise, we will expect to detect certain interactions between the components during relearning.

**Figure 4:**
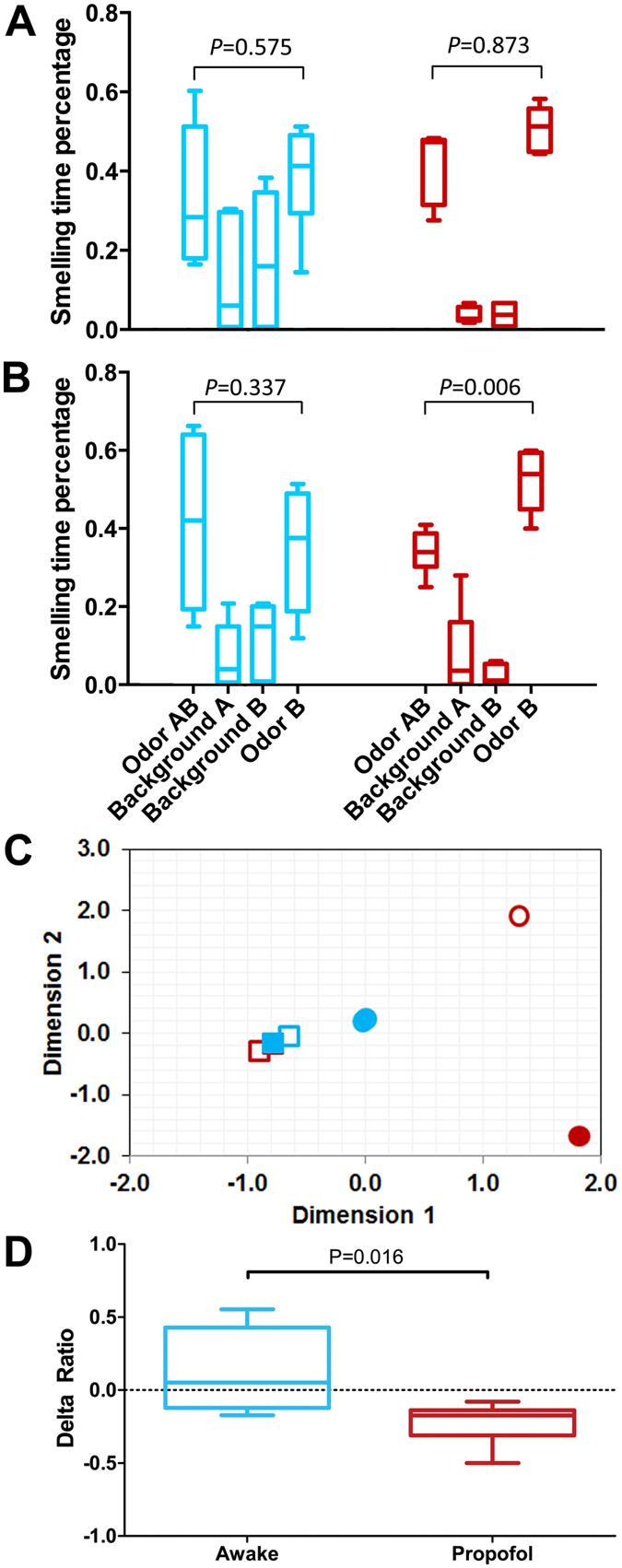
Anesthetic effects on relearning odor associations. (**A**) Percentage of time spent smelling a two-odor mixture (Odor AB) and one of the components (designated as Odor B), along with two background beads, during the first recognition test shown in Fig. 1. Odor mixture was previously exposed under awake (cyan) and propofol-anesthetized (red) conditions. Alternating components were used as Odor B in a counter-balanced design among different animals (n = 6). (**B**) Percentage of smelling time on the mixture (Odor AB) and one of the components (Odor B) during the second recognition test shown in Fig. 1, after animals consciously relearned the mixture along with one of the components. (**C**) Correspondence analysis of the discretized data from (**B**). Color scheme is the same as in (**A**,**B**). Solid and open circles are Odor AB and Odor B, respectively, and solid and open squares are Background A and B, respectively. (**D**) The delta ratios were calculated from (**B**) as (Odor AB − Odor B)/(Total Smelling Time). When the mixture was first learned under propofol anesthesia and relearned consciously with one of the components, the animals exhibited a memory deficit in recognizing the component but not the mixture. The same statistical method and presentation as in Fig. 3 are used.

Using the same learning and relearning protocol as depicted in Fig. 1, we first subjected new groups of animals to a 1:1 mixture of isoamyl acetate and decanal, either fully awake or fully anesthetized by propofol. Because the two odorants are structurally distinct, their sensory pathways before reaching the anterior piriform cortex are expected to have minimal overlaps^13^. We then allowed the animals to relearn the mixture consciously along with one of the pure components in the mixture in a counter-balanced design (*i.e*., one of the two components in the mixture was randomly selected as Odor B). As shown in Fig. 4, if the animals were awake during the first exposure to the mixture, they successfully learned the individual components in the mixture, as evidenced by the fact that both the mixture and the pure component were equally familiar (p = 0.575, 95% CI of difference [−0.143, 0.040]) to the animals during the second exposure (Fig. 4A), even though the pure component as a separate odor was presented to the animals for the first time. This conclusion is nontrivial because in other sensory systems, such as in the visual system, experiencing the mixture (*e.g.*, seeing yellow = red + green) is not necessarily equivalent to experiencing only one of the components (red or green).

The animals’ behavior became more complicated and interesting when the first exposure to the odor mixture was under propofol general anesthesia. As with a pure odorant, neither the mixture nor its components appeared familiar (p = 0.873, 95% CI of difference [−0.230, 0.031]) during the second exposure for conscious relearning (Fig. 4A). Notice the sharp difference between the times spent on the two background beads by the animals in the awake and propofol groups. Because the beads with the odor mixture and the odor component were unfamiliar to the propofol group, these animals spent significantly less time on the background beads as compared to the awake group. However, when tested after consciously relearning the mixture with one of the components, the animals in the propofol group exhibited a deficit in recognizing only the pure components but not the mixture. As shown in Fig. 4B, after conscious relearning, the animals in the propofol group spent significantly more time with the pure component than with the mixture (MD = −0.173, p = 0.006, 95% CI [−0.203, −0.158]). The correspondence analysis shows the times spent on the odor mixture and pure component as completely separated clusters for the propofol group (Fig. 4C), whereas the times spent on the background beads and on the scented beads by the awake group are clustered together (Fig. 4C). The delta ratios (Fig. 4D) show an opposite trend between the awake (MD = 0.051, 95% CI [−0.139, 0.470]) and propofol-anesthetized groups (MD = −0.173, 95% CI [−0.248, −0.158]), and the difference is highly significant (p = 0.016). This result indicates that for the animals in the propofol-anesthetized group, something about the mixture–in addition to simply coding its components–was novel during the conscious relearning. This “something” was not present during the first exposure under anesthesia. Consequently, the animals were able to relearn the mixture without showing the same deficit as with the pure odor component.

## Discussion

**Figure 5:**
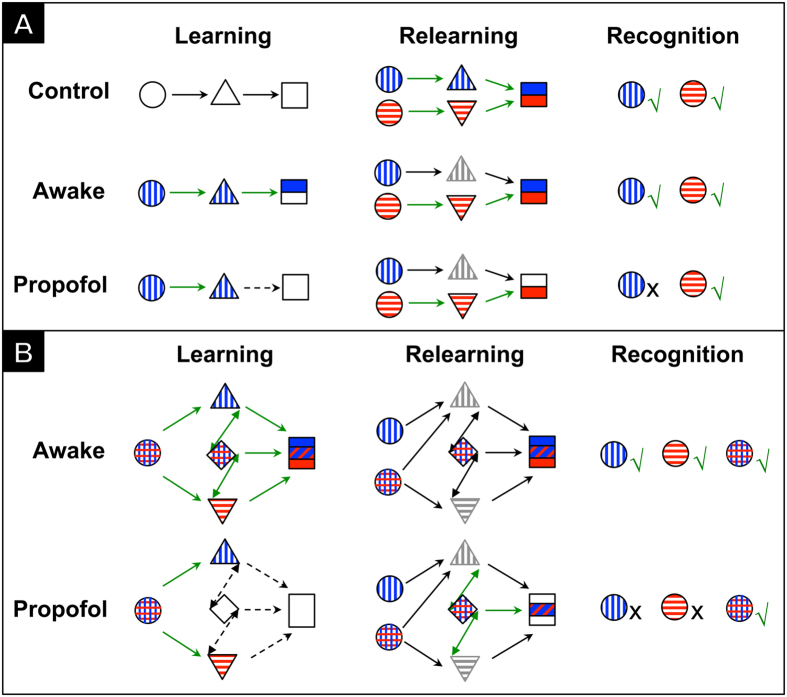
A possible mechanism of anesthesia-induced relearning deficits. Schematic representations of possible cellular processes in pure (**A**) and mixture (**B**) odor learning, relearning, and recognition. Circles, triangles, diamonds, and squares symbolize sensory input, cellular coding, cellular integration, and memory consolidation, respectively. Blue and red colors represent two distinct pure odorants and their separate anatomical coding pathways. Gray triangles indicate an absence of experience-induced Npas4 activation. Solid and broken arrows represent successful and unsuccessful cellular processes, respectively. Green and black arrows represent novel and familiar information processes, respectively. Odor coding under propofol anesthesia creates an unconscious cellular commitment that, while irretrievable, prevents future relearning of the same sensory information.

Our data revealed a rather complicated process with which general anesthesia causes anterograde amnesia and probably a prospective relearning deficit. Possible explanations of the results are schematically illustrated in Fig. 5, in which we use different colors and symbols to distinguish anatomical coding (triangles) from potential higher-order integration (diamonds) and consolidation (squares) involving other brain regions (*e.g.*, hippocampus and amygdala). When the animals’ cognitive memory of a pure odorant was tested after initial learning (Fig. 3A) and again after relearning alongside a competing pure odorant (Fig. 3B), the animals code and map the two odors independently, in agreement with previous findings^12^,^13^. Under general anesthesia, the transcriptional program for odor coding continues to occur, but is unable to reach consolidation. Because of the transcriptional modification, the anatomical loci are nevertheless occupied by the particular odor (gray triangles). These coding loci cannot be re-activated (Fig. 2B,C) during conscious relearning, leading to some hindrance to the animals to memorize the pure odor that has been first experienced under general anesthesia. Consequently, the animals are able to behaviorally remember and recognize Odor B but not readily recognize Odor A (Fig. 3D) after conscious relearning.

The results in Fig. 4 further substantiate that anesthesia-induced deficits in relearning do not occur at the stage of anatomical coding, but instead during integration and consolidation. Individual components in the odor mixture are likely coded successfully as with the pure odors during the first exposure, irrespective of whether the animals are awake or fully anesthetized. The fact that the animals in the propofol group fail to show familiarity with the odorants that have already completed transcription programming in the anterior piriform cortex suggests that memory consolidation, which involves communication of additional neurons from other brain regions, is blocked by propofol anesthesia. During relearning, the same set of neurons is targeted by the same odor mixture to reinforce the consolidation and integration in the awake group. For the anesthetized group, although the individual odor components are no longer novel for anatomical coding, the integration, which is blocked by anesthesia, remains novel during conscious relearning (Fig. 5B, lower row). As a consequence, the animals are able to consolidate and remember the association of the two odors in the mixture, but have difficulty remembering its pure components alone.

One of the hallmarks of general anesthesia is loss of consciousness. A recent theoretical work proposed a fundamental link between a phase transition for “information access” among multiple brain centers and the emergence of consciousness^14^. According to this theory, consciousness requires that sensory information, physically coded at a neuron or a brain center, be accessible by other neurons and brain centers in order for this information to percolate through an interconnected network of neurons for integration. The action of general anesthetics is to decrease the probability for any neurons or brain centers to communicate. In our experiments with an odor mixture, the physical coding of individual odorants reached specific neurons, but the accessibility to this coded information was blocked by propofol. For these animals, the association of coded information would be experienced for the first time during the relearning episode. Consistent with the neuroimaging finding that propofol reduced information integration and partial correlation^15^, our results provide behavioral evidence that information integration was absent during the initial odor exposure under propofol-induced unconsciousness.

Currently, there are two competing viewpoints regarding the recognition memory of a previously experienced stimulus. The first proposes that recognition memory consists of familiarity and recollection as two functionally distinct processes^7^, with the former being located in the perirhinal cortex and quickly accessible, and the latter being in the hippocampal and parahippocampal regions and requiring the contextual details of other associated information. The second, alternative viewpoint argues that familiarity and recollection are interrelated components of a single process without necessarily having a neuroanatomical separation and that the operational distinction between familiarity and recollection is merely weak versus strong memories due to cooperative and complementary functions of different medial temporal lobe structures^6^. The data in this study can help differentiate these two possibilities. In human familiarity tests, the objective is to quickly discriminate between studied and unstudied items by comparison. Thus, the forced-choice recognition test used in this study is most comparable to familiarity tests in humans, and our behavioral data reflect mainly the animals’ familiarity with, rather than recollection of, the odorants (i.e., the familiarity component of the recognition memory). The finding that recognition memories of an odor mixture and of its individual components can be manipulated differently by general anesthesia (Fig. 4D) seems to suggest that anesthesia blocks only the process for familiarity but not the process for recollection. Hence, it seems unlikely that familiarity and recollection are merely different strengths of a single process. However, after the anesthetized animals relearn the odor mixture with one of the components, they can quickly recognize the association of the mixture (recollection), but fail to display familiarity with its pure component (Fig. 4B,D), providing a counter-intuitive example that recollection can be accessed more quickly than familiarity. Moreover, because the commitment of physical loci for odor coding under general anesthesia does not affect recollection of association in odor mixture after conscious relearning, it might be overly simplistic to attempt to distinguish familiarity from recollection solely on the basis of neuroanatomical separation.

Clinically, our results would suggest that if a novel stimulation occurs under general anesthesia, an unconscious memory of this specific stimulation is possibly formed at the transcriptional level which, while irretrievable consciously, can nevertheless be manifested as selective blockade of future memory acquisition. This notion is entirely consistent with the conclusion from a recent study^16^, which shows that once an ensemble of neurons are allocated during the initial learning of a given event, the same ensemble is preferentially dedicated to the same learning and is not replaceable. Thus, if general anesthesia does not block the allocation of neuronal ensembles for the initial learning, as suggested by the Npas4 expression in Fig. 2, the allocated neuronal ensembles are histologically modified and consequently committed for that specific learning. The clinical implication of this finding warrants further investigation in humans, particularly in pediatric populations. For example, if the spelling of new words is recited under general anesthesia, will young patients later display certain difficulties memorizing those specific words, or is the association of letters in the sequence sufficient to forge the needed memory of correct spelling in a conscious relearning? Another implication of our finding is the potential of anesthesia-assisted neural programming for selectively blocking certain undesirable experience, such as in the case of treating addictions and substance abuse.

## Methods

### Animals

All animal experimental procedures were approved by the Institutional Animal Care and Use Committee at the University of Pittsburgh (Pittsburgh, PA, USA) and carried out in accordance with the approved guidelines. Seven-week-old male Sprague-Dawley rats were purchased from Harlan Laboratories (Indianapolis, IN) and were housed in a temperature and humidity-controlled room, with a 12-h light/dark cycle. Although the animals were not deprived of background odors in their continuously ventilated home cages and had free access to food and water, the odor environment in the dedicated housing facility was strictly controlled to be constant throughout the entire experiment period. Rats were randomly divided into control, awake, and propofol anesthesia groups and were handled identically except for the exposure to anesthesia and experimental odorants. For the anesthesia group, a surgical depth of general anesthesia was achieved by a 10-ml/kg intraperitoneal injection of propofol (10 mg/ml; Abbott Laboratories, Abbott Park, IL) and confirmed by sharp foot pinching or tail clamping before exposure to experimental odorants. Propofol was chosen because it is an odorless intravenous general anesthetic. The sealed vials and syringe prevented the slight phenolic odor of the emulsion from being exposed. For the awake group, 10 ml/kg normal saline was injected instead.

### Habituation and odor exposure

The odor exposure and behavioral test experiments were performed in a procedure room, which is separated from the housing room to prevent odor contamination. All animals were habituated to the behavioral testing environment 20 min per day for three days before any experiments. The testing chamber was situated inside a ducted fume hood, which vents directly to the exterior of the building. During habituation, unscented wooden beads (Woodworks Ltd., Haltom City, TX) and an empty, capless, 1.5-mL Eppendorf tube were placed inside the testing chamber as a part of the habituation conditions. In addition, four unscented wooden beads were placed inside the home cages during housing so that the animals became familiar with the beads. Each animal was given two of its own cage beads as the background beads in the forced-choice cognition tests (see below).

In the forced-choice behavioral tests, novel odors were provided to the animals in the form of scented beads. Reinforcement of learning and relearning for the immediate early gene response used the same odorant(s) in a 1.5-mL Eppendorf tube containing a small cotton ball, onto which a fixed amount of precisely diluted isoamyl acetate or decanal in mineral oil was added, as detailed previously^1^. Our previous pilot experiments showed that for steady-state odor delivery, this method is superior to the conventional proportional airflow method^17^, which is not only less precise depending on several physical factors of the odor delivery system, but is also susceptible to residual odor contamination in the gas manifold. The scented beads were prepared using the same procedure as described before^1^. Briefly, 100 μl pure odorants with a 1:10 dilution in mineral oil were injected into a cotton reservoir in the bottom of a 50-ml Falcon tube, which contained a spacer to prevent the wooden beads from directly contacting the scented oil. After carefully placing the beads above the spacer, the tube was tightly sealed until the beads were equilibrated with the odors. Our previous behavioral studies^1^ showed that the scented beads prepared this way had the ideal odorant intensity that was actively and directly investigated by rats. When the mixture of two novel odorants was used, the total odorant concentrations were kept the same so that the components in the mixture had the same odorant intensity as in the pure odorants.

The initial exposure to experimental odorants (*i.e.*, initial learning) and the re-exposure (*i.e.*, relearning) were 24 h apart. The odorant-containing Eppendorf tubes were introduced into the experimental chamber and the animals were allowed to freely sniff the tube for 20 min. For anesthetized animals, the odorants were introduced after the confirmation of deep anesthesia by sharp foot pinching or tail clamping (typically 15 min after the injection of propofol), and the distance between the odorant source and the nose was manually varied to mimic the movements of the awake animals^1^. Relearning was always performed consciously and involved two tubes: the previous learned pure odorant and another competing novel odorant, or the mixture of two pure odorants and one of the components in the mixture. The two odor-containing tubes were placed in the opposite corners of the experimental chamber during relearning.

### Forced-choice odor recognition tests

For different groups of animals, the forced-choice behavioral tests for odor recognition were conducted either 24 h after the initial learning or 24 h after the relearning. All tests were carried out with four choices: two scented beads and two background beads from the animals’ own home cage. The use of two background beads from the home cage allowed the assessment of the animals’ familiarity with the experimental odorants. The percentage of time spent on the background beads became significantly less when at least one of the scented beads was unfamiliar to the animals. The position of the two scented beads were randomly placed for each trial in the recognition tests. A video camera was placed outside the testing chamber to record the animals’ interactions with the beads. The first 5 min of the videos were reviewed and evaluated independently by two investigators blind of the experimental groups using the criteria for smelling behavior^1^. Smelling duration for all beads was summed and the time spent smelling each bead was presented as the percentage of the total smelling time to normalize for innately inquisitive animals. A delta ratio was calculated according to: Delta ratio = (T_OdorA_ − T_OdorB_)/(Total Smelling Time). A positive and negative delta ratio significantly different from zero suggests preference for Odor A and Odor B, respectively. A zero delta ratio means equal preference.

### Immunohistochemistry

The animals selected for histology measurements were sacrificed 1 h after the different odorant exposure procedures and perfused with phosphate-buffered saline (PBS) and a solution of 4% paraformaldehyde in PBS (pH 7.0). Brains were post-fixed overnight and cryo-protected in a 30% sucrose/PBS solution with 0.01% sodium azide. Sections in 30-μm thickness were generated using a sliding microtome. Primary antibodies against Npas4 (1:200; TeneTex, Inc., Irvine, CA) were incubated with free-floating sections overnight at 4 °C. Sections were washed with PBS three times, and then incubated with Alexa Fluor 488-conjugated secondary antibodies (1:500; Invitrogen, Carlsbad, CA) in darkness for 2 h at room temperature. Sections were then co-stained with the nuclear marker 4′,6-diamidino-2-phenylindole (1:1000; Sigma-Aldrich), washed, mounted onto slides and detected under fluorescence microscope. Images were acquired and analyzed using SlideBook software (Version 6, Intelligent Imaging Innovations, Inc., Denver, CO).

### Statistical analysis

The SPSS (IBM Corporation, Armonk, NY) and GraphPad (GraphPad Software, Inc., La Jolla, CA) programs were used for statistical analysis. Data from histology and behavioral measurements were first tested for normality and equality of variances. If null hypothesis for normality is accepted, one-way analysis of variance (ANOVA) followed by least significant difference (LSD) or Tamhane *post hoc* pairwise comparison was performed. Otherwise, data were either first ranked and compared using one-way ANOVA with LSD pairwise comparison on the ranked data, or analyzed using the non-parametric Kruskal-Wallis H test with pairwise comparisons. The forced-choice test results were also discretized by equal percentile binning, and group clustering was determined using correspondence analysis. The difference between smelling times on the two scented beads was tested for significance using the Wilcoxon signed-rank test, and the delta ratios were analyzed using ANOVA with LSD *post hoc* comparison. The confidence intervals at 95% were calculated by bootstrapping with 5000 samples using random seeds and the bias-corrected and accelerated procedures. All values are expressed and displayed using the box-and-whisker plots as median, first and third quartiles (box), and the full range (whiskers), unless otherwise noted. Statistical significance is assessed at a confidence interval level of 95% and a p < 0.05.

## Additional Information

**How to cite this article**: Jia, L.-J. *et al*. Effects of Propofol General Anesthesia on Olfactory Relearning. *Sci. Rep.* **6**, 33538; doi: 10.1038/srep33538 (2016).

## Figures and Tables

**Table 1:** Npas4^+^ Immunoreactivity in Layer II of Anterior Piriform Cortex.

Groups*	2σ above Mean Background Immunoreactivity†		
	Median	95% Confidence Interval	
		Lower	Upper
Control	3.0	1.6	5.2
Awake	94.5	92.3	98.5
Propofol	97.4	95.7	98.0
Awake-Awake	43.6	21.3	53.5
Propofol-Awake	39.6	8.7	65.0

^*^Groups are defined as in Fig. 2.

^†^σ: Standard deviation of the background Npas4 fluorescence intensity in the Control group.
